# 

**Published:** 1860-07-01

**Authors:** 


### CONTENTS.

| ART. | | PAGE |
|---|---|---|
| | Psychological Quarterly Retrospect | xli |
| I. | On the Reform of Lunatic Asylums | 277 |
| II. | A Medical Psychologist of the Seventeenth Century | 294 |
| III. | The Independence of the Soul | 314 |
| IV. | Notes on the Asylums of Italy, France, and Germany | 335 |
| V. | Dr. Laycock on Mind and Brain | 354 |
| VI. | The State of Lunacy in Scotland | 377 |
| VII. | Popular Physiology.—The Nervous System | 394 |
| VIII. | The Census of 1861 and Lunacy | 404 |
| | Foreign Psychological Literature | 407 |
| | Medico-Legal Trial | 433 |

